# Supplementary figures and images for: A combination of hepatic leukemia factor and circulating tumor cells serve as effective biomarkers for lung adenocarcinoma prognosis
Source: PeerJ. 2025 Mar 18;13:e19092. doi: 10.7717/peerj.19092 (PMC11927566; doi:10.7717/peerj.19092)

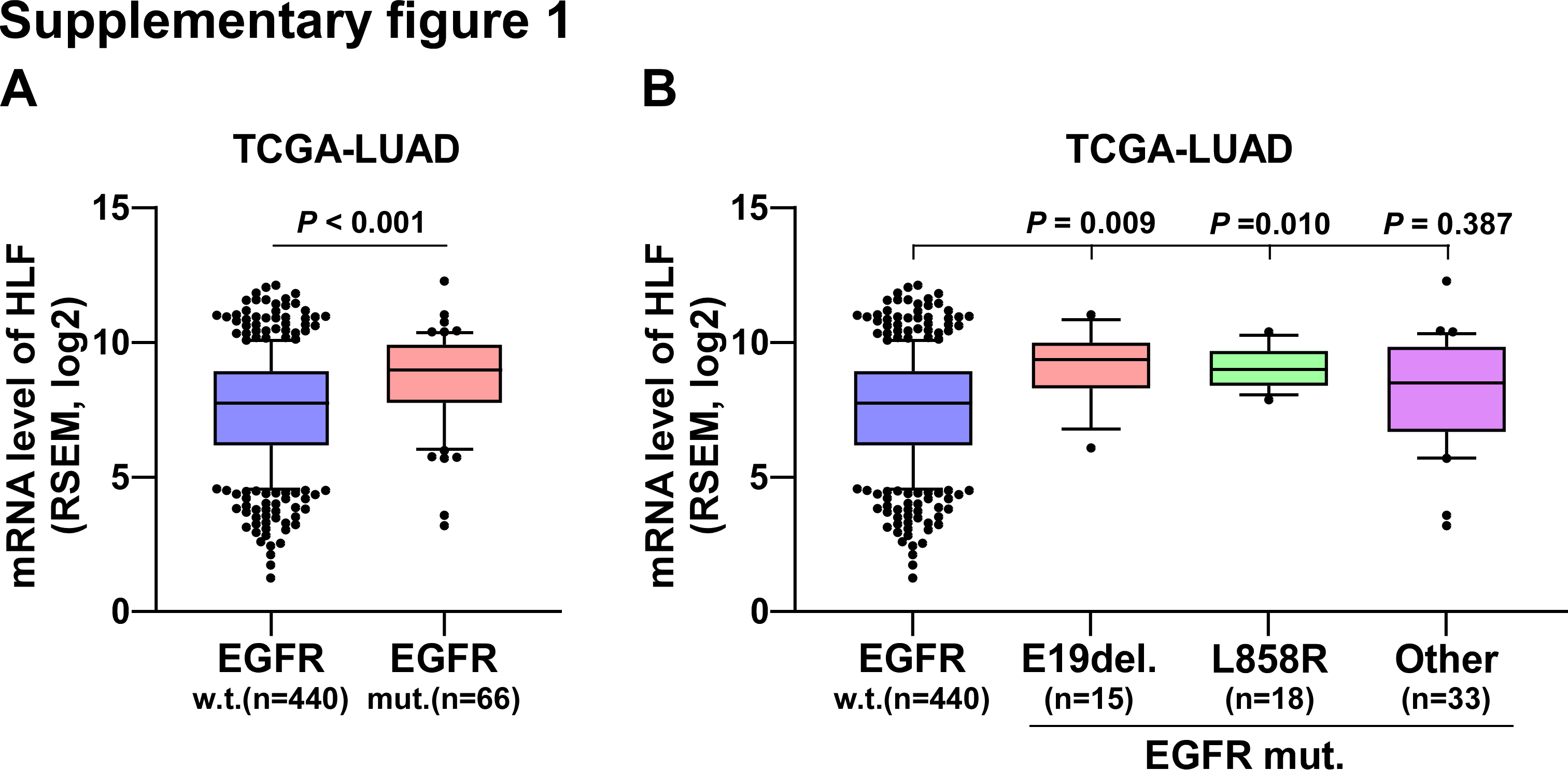

Supplement: Supplemental Information 1 — (A) mRNA expression of HLF in EGFR-mutated LUAD tissues from TCGA (w.t., n = 440; mut., n = 66;). (B) mRNA expression of HLF in different EGFR-mutated subtypes of lung ADC tissues from TCGA (w.t., n = 440; E19del., n = 15; L858R, n = 18; other, n = 33). Each bar represents the median values ± quartile values. P value was determined by one-way ANOVA test. [file peerj-13-19092-s001.png]

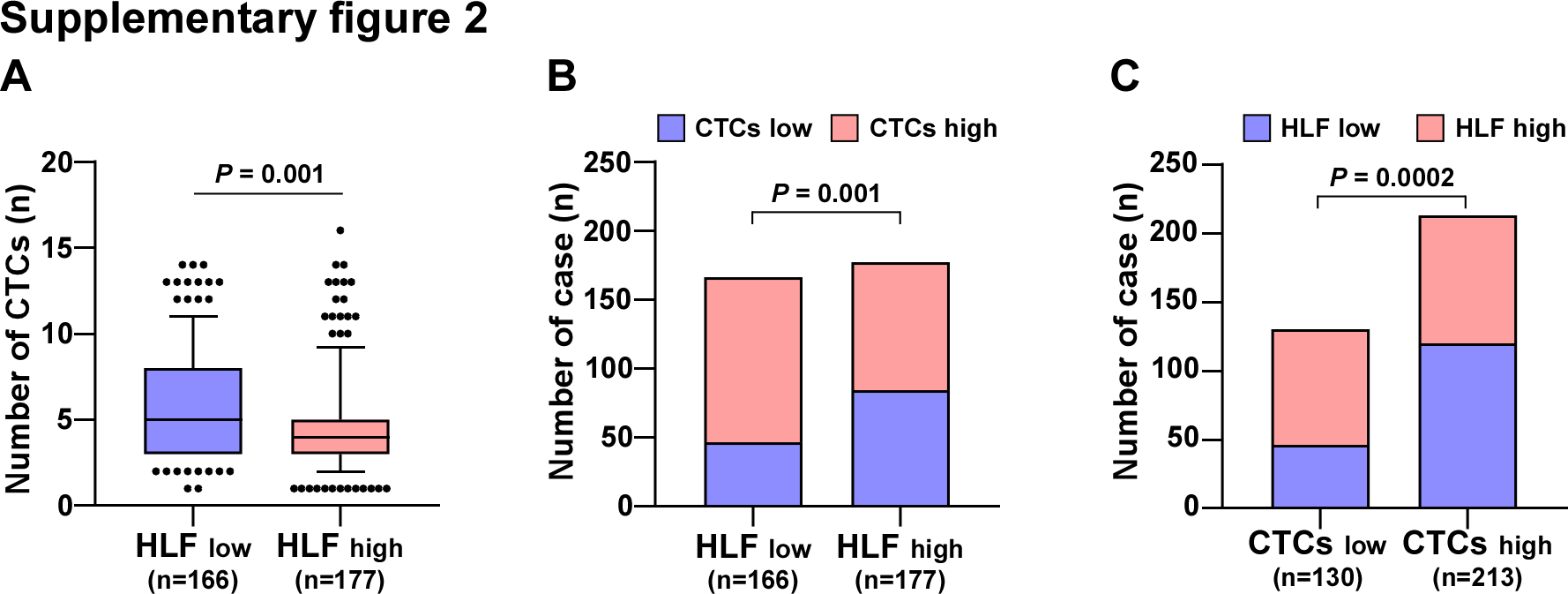

Supplement: Supplemental Information 2 — (A) CTCs number in HLF high expression LUAD patients compared with HLF low expression (HLF high., n = 177; HLF low, n = 166). Each bar represents the median values ± quartile values. P value was determined by one-way ANOVA test. (B) The ratios of CTCs low and CTCs high in LUAD patients with HLF high or low expression (HLF high, n = 177; HLF low, n = 166). P value was determined by one-way ANOVA test. (C) The ratios of HLF low and high in LUAD patients with CTCs high or low expression (CTCs high, n = 213; CTCs low, n = 130). P value was determined by one-way ANOVA test. [file peerj-13-19092-s002.png]
