# Supplementary material for: A combination of hepatic leukemia factor and circulating tumor cells serve as effective biomarkers for lung adenocarcinoma prognosis
Source: PeerJ. 2025 Mar 18;13:e19092. doi: 10.7717/peerj.19092 (PMC11927566; doi:10.7717/peerj.19092)
Supplement: Supplemental Information 3 [file peerj-13-19092-s003.docx]

##### Supplementary Table 1. The basic information of 343 patients with LUAD for analysis.

|  | | Cases (n) | Percentage (%) |
| --- | --- | --- | --- |
| Age | <60 | 121 | 35.3 |
|  | ≥60 | 222 | 64.7 |
| Gender | Female | 181 | 52.8 |
|  | Male | 162 | 47.2 |
| Grade | G1 | 42 | 12.2 |
|  | G2 | 200 | 58.3 |
|  | G3 | 101 | 29.4 |
| T classification | T1 | 133 | 38.8 |
|  | T2 | 153 | 44.6 |
|  | T3 | 36 | 10.5 |
|  | T4 | 16 | 4.7 |
|  | NA | 5 | 1.5 |
| N classification | N0 | 223 | 65.0 |
|  | N1 | 57 | 16.6 |
|  | N2 | 47 | 13.7 |
|  | N3 | 6 | 1.7 |
|  | NA | 10 | 2.9 |
| M classification | M0 | 318 | 92.7 |
|  | M1 | 25 | 7.3 |
| Stage | Stage I | 186 | 54.2 |
|  | Stage II | 67 | 19.5 |
|  | Stage III | 65 | 19.0 |
|  | Stage IV | 25 | 7.3 |
| EGFR mutation status | w.t. | 204 | 59.5 |
|  | mut. | 139 | 40.5 |

* LUAD: Lung Adenocarcinoma; NA: Not available; w.t.: wild type; mut.: mutation.

** EGFR mutation included: (1) 19del: p.E746_A750del (52 cases), p.E746_A750>RP (1 case), p.E746_S752>V (3 cases), p.E746_T751>A (1 case), p.E746_T751>VA (1 case), p.L747_A750>P (5 cases), p.L747_P753>S (3 cases), p.L747_T751del (4 cases). (2) p.L858R (56 cases). (3) Other: p.E746_A750del + p.L858R (2 case), p.E746_T751>A + p.L858R (1 cases), p.L861Q (1 case), p.H773_V774insNPH (1 case), p.V834L + p.L858R (1 case), p.E709A + p.G719S (1 case), p.G719A (4 cases), p.G719S (1 case), p.D770_N771insSVD (1 case).

##### Supplementary Table 2. The *EGFR* (18 ~ 21 exons) mutation model data reference Cosmic ID.

| Exon | | Cosmic ID | Amino acid sequence change | Base sequence changes |
| --- | --- | --- | --- | --- |
| 18 | | 6252 | G719S | 2155G>A |
|  |  | 6253 | G719C | 2155G>T |
|  |  | 6239 | G719A | 2156G>C |
| 19 | F | 26038 | K745_E749del | 2233_2247del15 |
|  |  | 28517 | E746_E749del | 2235_2246del12 |
|  |  | 13550 | E746_A750>IP | 2235_2248>AATTC |
|  |  | 6223 | E746_A750del (1) | 2235_2249del15 |
|  |  | 13549 | E746_T751>A | 2235_2251>AG(Complex) |
|  |  | 13552 | E746_T751>IP | 2235_2251>AATTC |
|  |  | 13551 | E746_T751>I | 2235_2252>AAT |
|  |  | 24869 | E746_T751delELREAT | 2235_2252del18 |
|  |  | 12385 | E746_S752>I | 2235_2255>AAT |
|  |  | 13557 | E746_A750>QP | 2236_2248>CAAC(Complex) |
|  |  | 12413 | E746_A750>RP | 2236_2248>AGAC(Complex) |
|  |  | 6225 | p.E746_A750delELREA(2) | c.2236-2250del 15 |
|  |  | 12728 | p.E746_T751delELREAT | c.2236-2253 del 18 |
|  |  | 12678 | p.E746_T751>A | c.2237-2251 del 15 |
|  |  | 12367 | p.E746_S752>A | c.2237-2254 del 18 |
|  |  | 12384 | p.E746_S752>V | c.2237-2255>T (Complex) |
|  |  | 18426 | p.E746_S752>V | c.2237_2256>TC |
|  |  | 12422 | p.L747_A750>P | c.2238-2248>GC (Complex) |
|  |  | 12419 | p.L747_T751>Q | c.2238-2252>GCA (Complex) |
|  |  | 23571 | p.L747_T751del | c.2238_2252del15 |
|  |  | 6220 | p.E746_S752>D | c.2238-2255 del 18 |
|  |  | 6218 | p.L747_E749delELREA | c.2239_2247delTTAAGAGAA |
|  |  | 12382 | p.L747_A750>P | c.2239-2248TTAAGAGAAG>C(Complex) |
|  |  | 12383 | p.L747_T751>P | c.2239-2251>C |
|  |  | 12420 | p.L747_T751>Q | c.2239_2252>CA |
|  |  | 6254 | p.L747_T751del | c.2239-2253 del 15 |
|  |  | 6255 | p.L747_S752delLREATS | c.2239-2256 del 18 |
|  |  | 12387 | p.L747_P753>Q | c.2239-2258>CA |
|  |  | 6210 | p.L747_T751>S | c.2240-2251 del 12 |
|  |  | 12369 | p.L747_T751del | c.2240-2254 del 15 |
|  |  | 12370 | p.L747_P753>S | c.2240-2257 del 18 |
|  | R | 26038 | K745_E749del | 2233_2247del15 |
|  |  | 28517 | E746_E749del | 2235_2246del12 |
|  |  | 13550 | E746_A750>IP | 2235_2248>AATTC |
|  |  | 6223 | E746_A750del (1) | 2235_2249del15 |
|  |  | 13549 | E746_T751>A | 2235_2251>AG(Complex) |
|  |  | 13552 | E746_T751>IP | 2235_2251>AATTC |
|  |  | 13551 | E746_T751>I | 2235_2252>AAT |
|  |  | 24869 | E746_T751delELREAT | 2235_2252del18 |
|  |  | 12385 | E746_S752>I | 2235_2255>AAT |
|  |  | 13557 | E746_A750>QP | 2236_2248>CAAC(Complex) |
|  |  | 12413 | E746_A750>RP | 2236_2248>AGAC(Complex) |
|  |  | 6225 | p.E746_A750delELREA(2) | c.2236-2250del 15 |
|  |  | 12728 | p.E746_T751delELREAT | c.2236-2253 del 18 |
|  |  | 12678 | p.E746_T751>A | c.2237-2251 del 15 |
|  |  | 12367 | p.E746_S752>A | c.2237-2254 del 18 |
|  |  | 12384 | p.E746_S752>V | c.2237-2255>T (Complex) |
|  |  | 18426 | p.E746_S752>V | c.2237_2256>TC |
|  |  | 12422 | p.L747_A750>P | c.2238-2248>GC (Complex) |
|  |  | 12419 | p.L747_T751>Q | c.2238-2252>GCA (Complex) |
|  |  | 23571 | p.L747_T751del | c.2238_2252del15 |
|  |  | 6220 | p.E746_S752>D | c.2238-2255 del 18 |
|  |  | 6218 | p.L747_E749delELREA | c.2239_2247delTTAAGAGAA |
|  |  | 12382 | p.L747_A750>P | c.2239-2248TTAAGAGAAG>C(Complex) |
|  |  | 12383 | p.L747_T751>P | c.2239-2251>C |
|  |  | 12420 | p.L747_T751>Q | c.2239_2252>CA |
|  |  | 6254 | p.L747_T751del | c.2239-2253 del 15 |
|  |  | 6255 | p.L747_S752delLREATS | c.2239-2256 del 18 |
|  |  | 12387 | p.L747_P753>Q | c.2239-2258>CA |
|  |  | 6210 | p.L747_T751>S | c.2240-2251 del 12 |
|  |  | 12369 | p.L747_T751del | c.2240-2254 del 15 |
|  |  | 12370 | p.L747_P753>S | c.2240-2257 del 18 |
| 20 | | 6241 | p.S768I | c.2303G>T |
|  |  | 12376 | p.V769_D770 ins ASV | c.2307_2308 ins(GCCAGCGTG) |
|  |  | 13558 | p.V769_D770insASV | 2309_2310AC>CCAGCGTGGAT(Complex) |
|  |  | 12378 | p.D770_N771insG | c.2310_2311ins GGT |
|  |  | 13428 | p.D770_N771insSVD | c.2311_2312insGCGTGGACA |
|  |  | 12377 | p.H773_V774insH | c.2319_2320ins CAC |
|  |  | 12381 | p.H773_V774insNPH | c.2319_2320insAACCCCCAC |
|  |  | 6240 | p.T790M | c.2369C>T |
| 21 | | 6224 | p. L858R | c.2573T>G |
|  |  | 6213 | p.L861Q | c.2582T>A |

* The EGFR (18 ~ 21 exons) mutation model data reference Cosmic ID.

(http://www.sanger.ac.uk/genetics/CGP/cosmic/)

##### Supplementary Table 3. The relationship between EGFR mutation status and clinical pathological characteristics in 343 patients with LUAD.

| Parameters | Number of cases | EGFR mutation status | | *P* values |
| --- | --- | --- | --- | --- |
|  |  | w.t. (n=204) | mut. (n=139) |  |
| Age |  |  |  |  |
| <60 | 121 | 65 | 56 | 0.109 |
| ≥60 | 222 | 139 | 83 |  |
| Gender |  |  |  |  |
| Female | 181 | 90 | 91 | <0.001 |
| Male | 162 | 114 | 48 |  |
| Grade |  |  |  |  |
| G1-G2 | 242 | 139 | 103 | 0.234 |
| G3 | 101 | 65 | 36 |  |
| T classification |  |  |  |  |
| T1 | 133 | 76 | 57 | 0.602 |
| T2-4 | 205 | 123 | 82 |  |
| N classification |  |  |  |  |
| N0 | 223 | 132 | 91 | 0.622 |
| N1-3 | 110 | 62 | 48 |  |
| M classification |  |  |  |  |
| M0 | 318 | 189 | 129 | 0.956 |
| M1 | 25 | 15 | 10 |  |
| Stage |  |  |  |  |
| I-II | 186 | 105 | 81 | 0.214 |
| III-IV | 157 | 99 | 58 |  |

* LUAD: Lung Adenocarcinoma.

##### Supplementary Table 4. The relationship between HLF expression level and clinical pathological characteristics in 343 patients with LUAD.

| Parameters | Number of cases | HLF expression level | | *P* values |
| --- | --- | --- | --- | --- |
|  |  | low (n=166) | high (n=177) |  |
| Age |  |  |  |  |
| <60 | 121 | 49 | 72 | 0.031 |
| ≥60 | 222 | 117 | 105 |  |
| Gender |  |  |  |  |
| Female | 181 | 90 | 91 | 0.603 |
| Male | 162 | 76 | 86 |  |
| Grade |  |  |  |  |
| G1-G2 | 242 | 106 | 136 | 0.008 |
| G3 | 101 | 60 | 41 |  |
| T classification |  |  |  |  |
| T1 | 133 | 50 | 83 | 0.003 |
| T2-4 | 205 | 111 | 94 |  |
| N classification |  |  |  |  |
| N0 | 223 | 88 | 135 | <0.001 |
| N1-3 | 110 | 70 | 40 |  |
| M classification |  |  |  |  |
| M0 | 318 | 147 | 171 | 0.004 |
| M1 | 25 | 19 | 6 |  |
| Stage |  |  |  |  |
| I-II | 186 | 67 | 119 | <0.001 |
| III-IV | 157 | 99 | 58 |  |

* LUAD: Lung Adenocarcinoma.
